# The evolving landscape of brain metastasis: volume II

**DOI:** 10.1016/j.trecan.2025.11.007

**Published:** 2025-12-02

**Authors:** Manuel Valiente, Carey Anders, Adrienne Boire, Benjamin Izar, Nuria Kotecki, Srinivas Malladi, Joan Massagué, Nelson S. Moss, Josh Neman, Matthias Preusser, Sanne Schagen, Peter M. Siegel, Hussein Tawbi, Varun Venkataramani, Frank Winkler, Gelareh Zadeh, Johanna A. Joyce

**Affiliations:** 1 Brain Metastasis Group, Spanish National Cancer Research Centre, Madrid, Spain; 2 Duke Cancer Institute, Durham, NC, USA; 3 Memorial Sloan Kettering Cancer Center, New York, NY, USA; 4 Columbia University Irving Medical Center, New York, NY, USA; 5 Institut Jules Bordet, Hôpital Universitaire de Bruxelles, Brussels, Belgium; 6 Department of Pathology, UT Southwestern Medical Center, Dallas, TX, USA; 7 Department of Neurosurgery and Brain Metastasis Program, Memorial Sloan Kettering Cancer Center, New York, NY, USA; 8 Department of Neurological Surgery, Keck School of Medicine, University of Southern California, Los Angeles, CA, USA; 9 Division of Oncology, Department of Medicine I, Medical University of Vienna, Vienna, Austria; 10 NKI, Amsterdam, The Netherlands; 11 Goodman Cancer Institute, Department of Medicine, McGill University, 1160 Pine Avenue, West, Montreal, Quebec H3A 1A3, Canada; 12 Melanoma Medical Oncology, UT MD Anderson Cancer Center, Houston, TX, USA; 13 Heidelberg University Medical Faculty, Neurology Clinic, University Hospital Heidelberg, and European Center for Neurooncology, Heidelberg, Germany; 14 German Cancer Research Center, Heidelberg, Germany; 15 Mayo Clinic, Rochester, NY, USA; 16 University of Lausanne, Ludwig Institute for Cancer Research, Lausanne, Switzerland

## Abstract

Brain metastasis (BrM) represents the most common intracranial malignancy, arising in up to 30% of all adult cancer patients and contributing significantly to cancer-related morbidity and mortality. BrM is now recognized as a biologically distinct condition with unique mechanisms of organotropism, colonization, and therapeutic vulnerability. We highlight recent progress in omic and spatial profiling, which has revealed key drivers of brain tropism. These findings have reshaped therapeutic strategies, leading to clinical trials that specifically address central nervous system (CNS) involvement. Emerging approaches now include efforts to prevent brain relapse. Preclinical models increasingly provide sophisticated platforms to evaluate next-generation therapies. Collectively, these advances are transforming the clinical landscape, offering new hope for the prevention and management of BrM through precision medicine and integrated therapeutic strategies.

## Introduction

BrMs represent the most common intracranial malignancy and are a major source of morbidity and mortality across solid cancers. Once regarded as the end-stage manifestation and inevitable complication of systemic disease, BrMs are now recognized as a biologically distinct condition with unique molecular drivers, microenvironmental interactions, and clinical vulnerabilities. To facilitate research, dedicated consortia and institutional initiatives were established (Box 1), Over the past decade, discoveries at the interface of cancer biology, neuroscience, and immunology, together with important therapeutic advances have begun to reshape patient outcomes.

**Figure 1.**
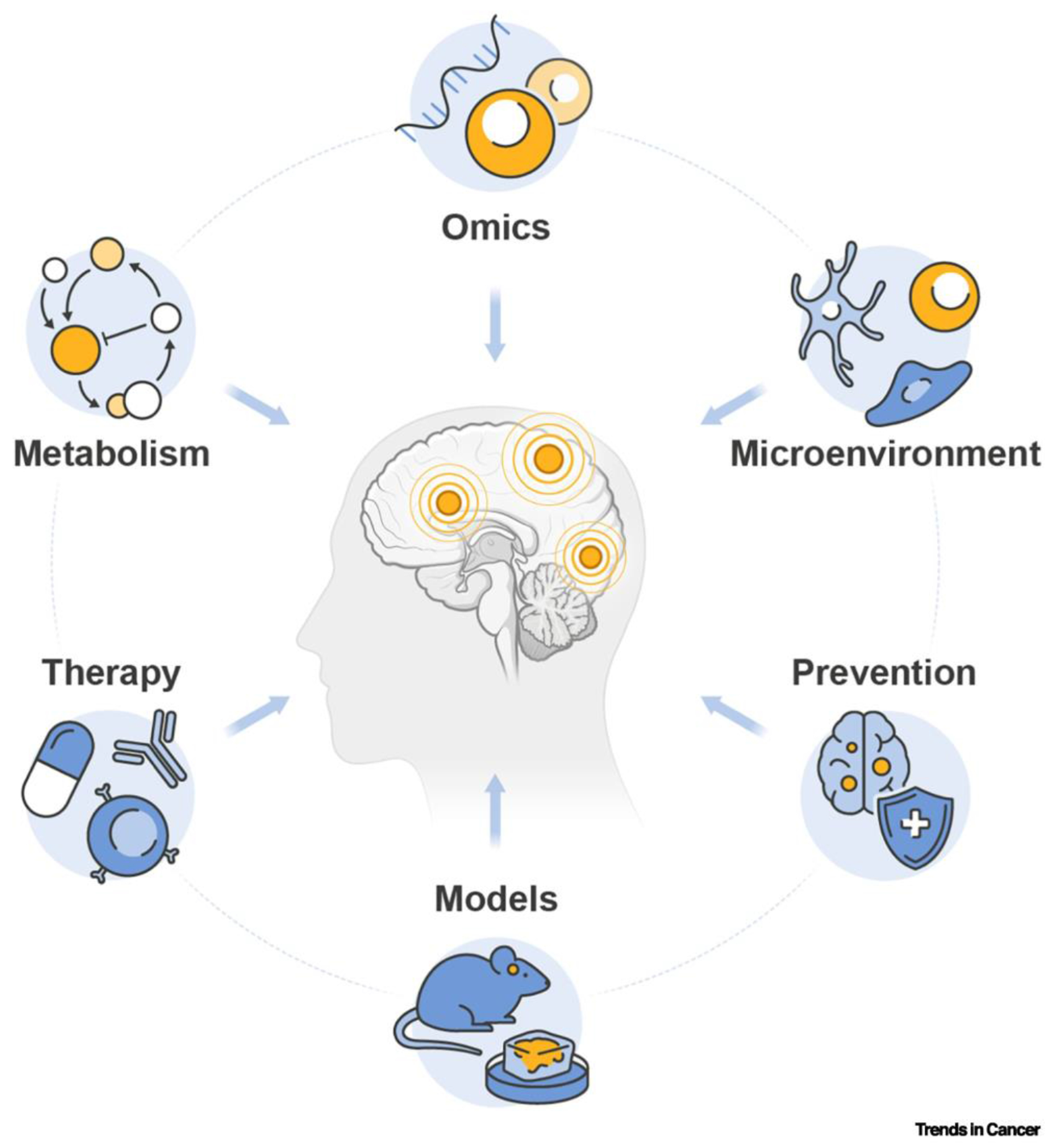
The evolving landscape of brain metastases. The scheme represents the areas discussed in this review (Volume II). The main areas of progress in brain metastases, as identified by the coauthors, include: (i) omic approaches implemented in a steadily growing number of human brain metastases, especially related to single-cell approaches; (ii) the biology of specific compartments of the microenvironment, in particular the immune system, astrocytes, and endothelial cells; (iii) the emerging research on the metabolic vulnerabilities of metastasis and metabolic adaptations specific to brain metastases; (iv) the promising results on biomarkers that could predict the development of brain metastases from primary sources as well as liquid biopsies; (v) the improvements in experimental modeling as well as the development of technologies for preclinical and clinical use; and (vi) the impressive number of new systemic therapies capitalized on by immunotherapy, antibody–drug conjugates, and small molecules.

The aim of this review is to provide an updated synthesis of these developments, integrating insights from genomics, single-cell and spatial technologies, neuroimmunology, cancer neuroscience, and metabolism with translational advances in modeling, prevention, and therapy (Figure 1). As highlighted throughout this review, a new era is emerging – one that recognizes BrM as not only a biologically distinct but also a treatable, and in some cases preventable, manifestation of systemic cancer. Nonetheless, these advances have also illuminated remaining challenges and areas primed for further discovery.

This is a timely moment for such a synthesis: the increasing inclusion of patients with BrM in clinical trials, the availability of predictive biomarkers and experimental models, and the establishment of international research consortia have positioned the field for rapid progress. By linking biological mechanisms with clinical innovation, our goal is to outline the evolving landscape of BrM and identify opportunities to accelerate discoveries that improve patient care.

## Insights from orthogonal omics strategies

Comprehensive genomic profiling studies have provided critical insights into the molecular landscape of BrM. Comparative analysis of BrMs with matched primary tumors or extracranial metastases have revealed distinct evolutionary paths and uncovered features driving brain-metastatic organotropism. More recently, the emergence of single-cell and spatial omics technologies has revolutionized this field [1–5], enabling high-resolution dissection of tumor ecosystems and uncovering cellular programs and interactions masked in bulk analyses. Single-cell RNA-seq (scRNA-seq) and single-nucleus (sn) sequencing approaches have uncovered phenotypic diversity among cancer, immune, and stromal cells, while spatially resolved platforms – such as spatial transcriptomics and multiplexed protein imaging – preserve tissue architecture, allowing precise mapping of cell–cell interactions and microenvironmental niches. Together, these technologies provide complementary perspectives on the transcriptional, cellular, and spatial organization of BrMs, illuminating both genetic and non-genetic mechanisms of brain colonization. In this section, we review some of the most impactful findings from recent patient-based studies that leverage these orthogonal approaches.

### Recent advances in the genomics of BrM

Following a landmark study [6] proposing the branched evolution hypothesis of BrM [7], multiple studies have expanded this work across several cancer types, including non-small cell lung cancer (NSCLC) [5,8–13], breast cancer [14–18], and melanoma [19–24], the major primary tumors that seed metastases to the brain. Collectively, these studies now include hundreds of human BrMs derived from diverse primary tumors, with over 200 samples matched to primary tumors or extracranial metastasis counterparts. Notably, these studies are increasingly associated with a number of publicly available resources for the BrM community.

Several recurring genomic findings have emerged. High frequencies of *CDKN2A/B* loss, *PIK3CA* mutations, and *TP53* mutations or loss in BrM have been consistently reported across multiple studies. It remains largely unclear; however, whether these divergent mutations drive brain-metastatic organotropism or reflect broader prometastatic genomic alterations. The potential therapeutic benefit of tailored targeted strategies is yet to be examined. Further efforts are required to increase our limited knowledge on the genomic landscape of BrMs from colorectal cancer as well as renal cancer, which are increasing in relative incidence [25]. Lastly, while small-cell lung cancer (SCLC) has a very high BrM incidence (~80%), no matched genomic data have been reported to date. This gap underscores the challenges of studying BrM biology in clinical contexts that do not routinely require biopsy or resection of the primary tumor and corresponding BrM.

### Chromosomal instability (CIN) as a potential hallmark of BrM

There is increasing evidence indicating that BrMs are more chromosomally unstable than primary tumors and extracranial metastases. CIN, a hallmark of cancer characterized by frequent changes in chromosome number and structure, often arises from mitotic segregation errors. CIN is linked not only to metastasis but also to immune evasion and therapy resistance, suggesting a broader role in shaping metastatic fitness. A frequently used molecular profiling proxy of CIN is the fraction of genome altered (FGA). At least six recent studies [5,9–12,26] have shown that BrMs exhibit significantly higher rates of CIN, as inferred from FGA measurements or using an RNA-based signature (*CIN70*), compared with matched/unmatched primary tumors or extracranial metastases. These findings span multiple cancer types, including melanoma, NSCLC, colorectal, and breast cancer, among others. This is supported by experimental evidence, quantifying micronuclei that arise from CIN, in melanoma and NSCLC, including matched specimens of primary tumors and BrMs [12].

Intriguingly, CIN-high human cancer cell lines show increased brain-metastatic potential in experimental models, suggesting that CIN may mechanistically contribute to brain-specific organotropism [5]. CIN levels in primary tumors have also been found to predict subsequent development of BrMs [12]. Although subsets of patients with treatment-naïve BrMs were profiled at the time of diagnosis in these studies, frequently with the brain being the first clinically sampled site of disease, it remains unclear whether CIN actively promotes brain tropism or rather evolves more rapidly than primary tumors and extracranial metastases, potentially in response to the unique barriers and selective pressures of the brain microenvironment.

### Identification of cellular states with single-cell approaches

The advent of single-cell genomic methods, namely sc/snRNA-seq and spatial transcriptomics, along with analytical advances, have been instrumental in dissecting metastatic ecosystems [13]. In addition to exploring the complexity of the metastasis-associated microenvironment, single-cell approaches enable the resolution of intrametastasis heterogeneity and identification of cancer cell subpopulations. Currently, the most comprehensive data exist for BrMs arising from NSCLC. For example, scRNA-seq analyses of ten NSCLC BrMs were reported [27], identifying loss of bronchoalveolar differentiation states as a shared hallmark among malignant cells in the brain. A recent study [12] reported a comprehensive analysis of 31 NSCLC BrMs compared with 12 primary tumors (including matched samples) profiled by snRNA-seq, whole-genome sequencing (WGS), and spatial transcriptomics. This uncovered a pronounced loss of lineage differentiation in most cancer cells. Intriguingly, cancer cells with neural-like features resembling those of primary brain tumors (e.g., glioma) pre-existed in primary tumors but were strongly enriched in BrMs, raising the possibility that these cells may be prone to brain tropism [12]. These observations were confirmed in an independent set of 18 pairs of primary tumors and matched BrMs [12].

Consistent with these findings, single-cell transcriptomic analyses of melanoma patients also revealed that brain-metastatic melanoma cells adopted a neural-like state compared with extracranial melanoma metastases [5]. In this study, upregulation of neural-cell adhesion factor 1 (NCAM1) was confirmed by protein staining in an independent set of 40 melanoma patients [5]. This reinforces the notion that neural-like states are an important transcriptional and phenotypic hallmark of BrM across cancer types. Spatial transcriptomics data in NSCLC suggest that brain-tropic cancer cells adopt neural-like states early in their development, potentially even before extravasation across the blood–brain barrier (BBB). These pre-adapted cancer cells subsequently engage in specific interactions with tumor-infiltrating or tissue-resident myeloid cells on arrival in the brain. Conversely, immune cells – particularly myeloid populations – also shape cancer cell programs, influencing key pathways such as the epithelial-to-mesenchymal transition (EMT) and MYC signaling [12].

A recent study [26] reported scRNA-seq of >100 BrMs and clinically matched primary tumors across cancer lineages. Samples included NSCLC – comprising 20 epidermal growth factor receptor (EGFR)-mutant tumors and therefore distinct from another recent report [12] – different subtypes of breast cancer, melanoma, colorectal cancer, and others. This analysis confirmed multiple prior observations, such as an increased CIN rate in BrMs and a more immunosuppressive microenvironment [28–30]. While expression of specific genes varied in different lineages, this study [26] also found recurring neural-like differentiation of malignant cells from BrMs across cancer lineages.

## Tumor microenvironment (TME)

**Figure 2.**
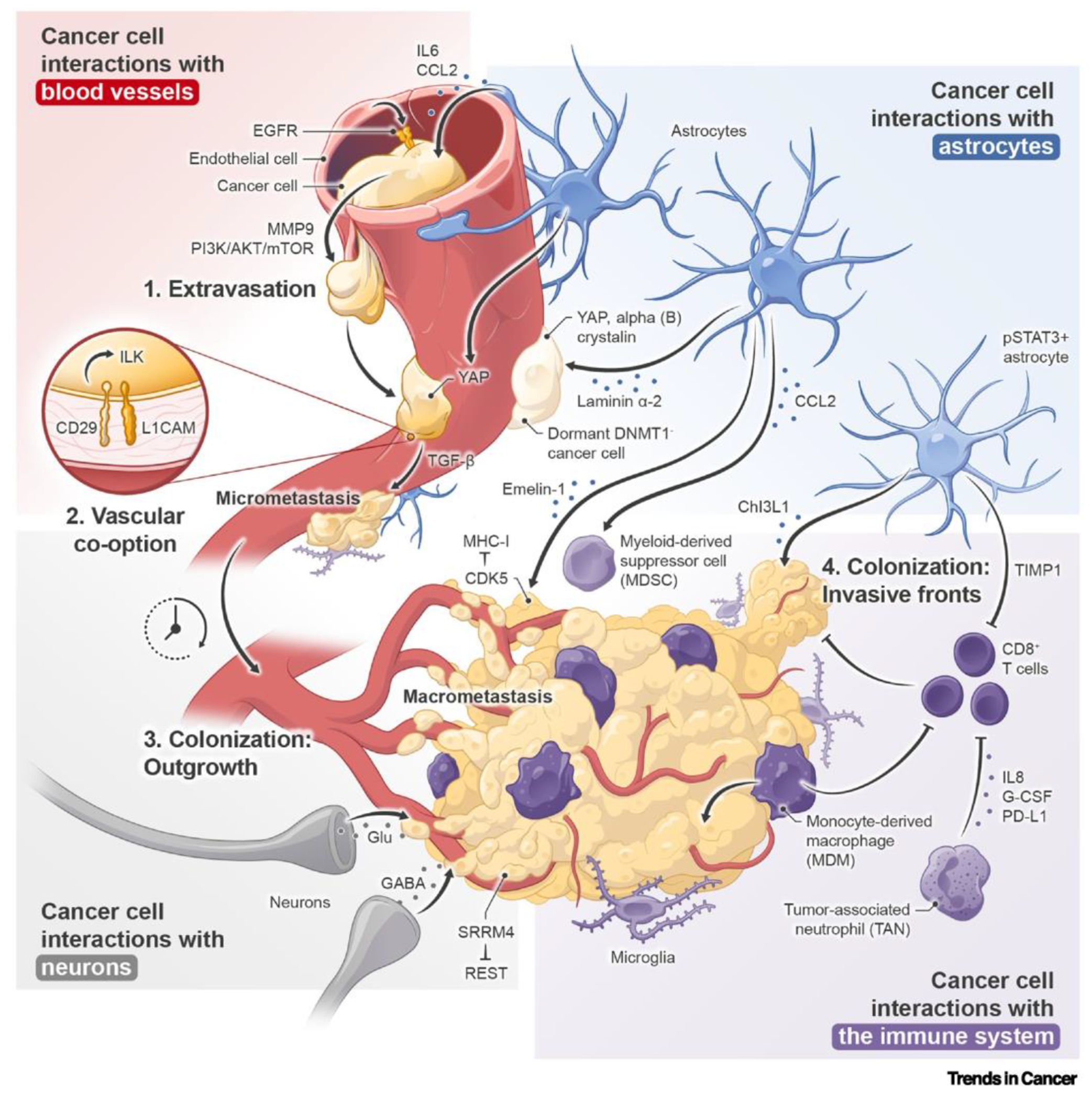
Brain metastasis microenvironment. The scheme represents the temporal steps of micrometastases (left side) and macrometastases (right side) as well as the specific crosstalk with the major microenvironment components, focused on blood vessels, astrocytes, and the immune system [including T cells, neutrophils, and myeloid-derived suppressor cells (MDSCs)]. We also include the emerging crosstalk with neurons, although this is a topic that requires further research (see later). Knowledge on the crosstalk between metastatic cells and each specific cell type of the microenvironment has evolved beyond describing molecular mediators. Aspects such as validating their functional contribution *in vivo*, defining the crosstalk within the microenvironment and/or cancer cells, and dissecting disease-associated subpopulations (pSTAT3^+^ reactive astrocytes) could be used to further develop targeted strategies and/or be the source of novel biomarkers with clinical value. All this progress is also being translated into novel clinical trials that may lead to interventions that impair the progression of metastases in the brain by targeting specific compartments of the microenvironment. Research focused on the metastasis-associated microenvironment additionally offers a dynamic picture where cancer cells, specific cell types from the brain, or those that infiltrate the brain, modify their phenotypes as they coevolve with the metastases. Finally, although still marginally studied in the context of metastases, the crosstalk between metastatic cells and neurons involves the ability of metastases to use key neurotransmitters as part of their metabolic adaptation, but such crosstalk might also interfere with neuronal communication and brain function, which may underly neurocognitive deficits in patients. In the figure, some functional mediators of the crosstalk with the brain metastasis microenvironment are described. However, this figure is not intended to provide a comprehensive review of the brain metastasis microenvironment bibliography. Instead it should be used as a focused update on specific cell types of the microenvironment interrogated by multiple publications in the past 8 years and the limited space available to cover a more comprehensive analysis. Abbreviations: EGFR, epidermal growth factor receptor; PD-L1, programmed death-ligand 1.

The brain microenvironment is unique relative to other metastatic sites, due to the distinctive composition of the extracellular matrix, the presence of the BBB, and tissue-resident cell types that include neurons, microglia, oligodendrocytes, and astrocytes [31]. In this section, we review recent advances in understanding how specific components of the brain microenvironment, which include the vasculature, astrocytes, and the immune system, contribute to the formation of BrMs originating from diverse solid cancers (Figure 2). When appropriate, we highlight potential avenues for clinical translation. We also note the currently limited published work on the crosstalk between brain-metastatic cancer cells and neurons, despite the well-known impact that BrMs can have on neurological function. The latter subject promises to be an intensively studied research area in the coming years.

### Crosstalk with the vasculature

The BBB comprises endothelial cells with robust tight junctions that are surrounded by pericytes/mural cells and astrocyte foot processes, creating a barrier that protects the brain [32]. To seed this organ, disseminated cancer cells must first traverse the BBB. Post-extravasation, cancer cells retain interactions with the perivascular niche to successfully colonize the brain.

#### Extravasation.

Research on the complex mechanism of extravasation through the BBB continues to uncover new findings. Brain endothelial cells may not be a passive compartment in this process as they have been shown to produce factors that activate EGFR signaling in breast cancer cells, facilitating morphological changes required for BBB extravasation [33]. In addition, while cancer cells are still in an intravascular position, PI3K/AKT/mTOR signaling is activated [34] and cancer cell-derived MMP9-mediated remodeling of endothelial cells facilitates their extravasation into the brain parenchyma [35]. Extravasation is a critical step for brain colonization and thus it holds great promise therapeutically; however, how to target this step in the metastatic cascade in a clinically compatible context remains unclear.

#### Vessel co-option.

After traversing the BBB, disseminated cancer cells frequently remain associated with the abluminal side of the vessel, performing vascular co-option [7]. This process, which involves L1CAM engagement, also stimulates ILK-dependent signaling downstream of integrin β1 in cancer cells, driving morphological changes that enable cancer cells to spread along the vasculature [36]. Cell spreading along the vessels involves YAP nuclear translocation and the activation of transcription programs that promote cancer cell proliferation [36]. Interestingly, different growth patterns have been described during the initiation of BrMs. Her2^+^ breast cancer cells form more compact nests driven by the expression of tenascin C. The organization of these metastases excludes astrocytes and microglia from the tumor core, resulting in their distribution towards the periphery of these lesions [37]. By contrast, triple-negative breast cancer (TNBC) cells typically maintain close association with the vasculature and exhibit vessel co-option, with diffuse contacts to astrocytes and microglia [37]. Whether these early cellular interactions dictate the persistent growth patterns observed in BrMs from various solid cancers remains an open question [38,39].

This type of non-angiogenic interaction with the vasculature expands beyond the early stages of brain colonization as metastatic cells can migrate and invade through the brain along vessels. Perivascular invasion is readily observed in BrMs, particularly those of melanoma origin [40]. Melanoma cells exhibiting PTEN loss readily undergo perivascular invasion in response to endothelially derived TGFβ [41]. This process is initially supported by the increased stiffness of the subendothelial basement membrane relative to the softer brain parenchyma, which promotes the formation of focal adhesions in melanoma cells, enhancing perivascular migration [42].

Together, these findings underscore the importance of reciprocal interactions between cancer cells and endothelial cells during several steps of organ colonization and emphasize the relevance of non-angiogenic interactions that are yet to be exploited therapeutically.

### Crosstalk with astrocytes

Astrocytes have progressively emerged as a critical brain-resident cell type, playing complex roles during BrM formation. Recent findings continue to expand their roles associated with BrM.

#### Alteration of vasculature integrity by astrocytes.

As part of the BBB or the blood–tumor barrier (BTB), astrocyte-derived secreted factors (e.g., IL-6, CCL2) can positively influence the initial seeding of cancer cells by increasing BBB permeability or by directly stimulating the extravasation of cancer cells across an intact BBB [43].

#### Astrocyte-mediated dormancy of metastatic cancer cells.

Post-extravasation, reactive astrocytes can influence the fate of cancer cells that colonize the brain. Factors secreted by astrocytes were shown to suppress DNMT1 expression in cancer cells *in vitro*. Moreover, DNMT1 silencing was associated with elevated expression of genes encoding L1CAM and aB-Crystallin, which enable cancer cell survival and entry into a dormant state following seeding in the brain [44]. Complementarily, BrM cells that are ensheathed by astrocyte endfeet exist in a dormant state, while proliferative micrometastases exhibited reduced endfoot coverage [45]. Furthermore, astrocyte-derived laminin-a2 induced the expression of dystroglycan in breast cancer cells, resulting in cytoplasmic retention of YAP and establishment of the dormant state [45]. These studies reveal that astrocytes may play a central role in regulating metastatic dormancy.

#### Emergence of astrocytes as modulators of local immunosuppression.

The complex crosstalk between astrocytes and cancer cells includes astrocyte subsets that contribute to the growth of metastases through impairment of antitumor immune responses. This subset of reactive astrocytes was identified by the activation of STAT3, and pSTAT3^+^ astrocytes enhanced the formation of BrM, in part, due to their ability to locally suppress activated CD8^+^ T cells [46] by secreting TIMP1 [47], a STAT3 target gene. Consequently, increased T cell infiltration and activation was demonstrated on ablation of TIMP1 specifically in astrocytes, which resulted in reduced BrM [47]. Interestingly, an orally available STAT3 inhibitor was shown to have antitumor efficacy in patients with BrMs under compassionate use, which is now in clinical trials (NCT05689619), suggesting that targeting key stromal populations in the BrM microenvironment could be an effective strategy [46]. Additional findings reinforce a role for astrocytes in favoring the escape of metastatic cells from T cell-mediated killing. For instance, downregulation of class I MHC in metastatic cells has been shown to be triggered by astrocyte-derived Emelin-1 [48]. Emelin-1 induces the expression of CDK5 in breast BrM cells, which downregulated MHC-I [48]. Furthermore, metastasis-associated astrocytes also promote local immunosuppression by enriching specific compartments of the innate immune system. For instance, monocytic myeloid-derived suppressor cells (M-MDSCs) are attracted to BrMs due to the secretion of CCL2 by astrocytes [49], and LCN2-expressing granulocytes also promote metastasis outgrowth in the brain after being recruited from the bone marrow in an astrocyte-dependent manner [50]. The emerging role of astrocytes in modulating the immune landscape to favor an immunosuppressive microenvironment may open opportunities for targeting reactive astrocytes (e.g., STAT3 inhibitors); for example, in combination with immune checkpoint inhibitors [51]. Such a strategy could provide additional benefits as the STAT3 subpopulation of astrocytes is also enriched in highly invasive BrMs from a variety of cancers [38]. Astrocyte-specific ablation of STAT3 resulted in diminished BrM growth in mice and impaired cancer cell invasion in a chitinase-3-like-1 (ChI3L1)-dependent manner, which was identified as a STAT3-dependent key driver of BrM cell invasion [38].

#### Astrocytes are functionally reprogrammed by cancer cells.

The protumoral phenotypes associated with astrocytes in BrM seem to originate by the increasing levels of factors secreted by cancer cells, which would trigger the functional reprogramming of intrinsically plastic glial cells. For instance, astrocytes are influenced by SCLC cells to adopt a transcriptional program that includes genes associated with the neuroprotective functions of astrocytes (NGF, BDNF, TGF-a, SERPINE1). Interfering with this program, which is activated in astrocytes, resulted in increased cancer cell death and reduced metastatic burden [52].

### Crosstalk with the immune system

The healthy brain maintains a tightly regulated inflammatory environment to prevent excessive neuroinflammation. In the context of cancer, this balance is disrupted, leading to a dynamic interplay between mechanisms that suppress inflammation to preserve brain function and those that promote antitumor immunity. Immune landscapes in BrM are notably more diverse relative to primary brain tumors, as revealed by recent analyses of the brain TME in patient samples [2,28–30,53,54]. While the immune system plays a central role in regulating metastatic progression in peripheral tissues, the brain presents a unique immunological environment where tightly controlled inflammatory responses often limit effective antitumor immunity. BrMs are infiltrated by tumor-associated macrophages (TAMs), including monocyte-derived macrophages (MDMs) and microglia, various T cell populations, neutrophils, and other immune cells. Many of these cell types can exert immunosuppressive functions, contributing to a permissive environment for metastatic seeding and growth.

#### T cell subsets and antitumor immunity in BrMs.

T cells can infiltrate BrMs by distinct anatomical routes [55] but to varying degrees, with significant interpatient heterogeneity in their abundance and functional states [28,30]. While some BrMs, particularly those of lung cancer origin (NSCLC, lung adenocarcinomas), harbor CXCL13-expressing CD39^+^ T cells that are potentially tumor reactive (pTRT), most lesions exhibit relatively low T cell infiltration and limited tumor reactivity [30]. Even in pTRT cell-high tumors, where clonally expanded CD8^+^ T cells suggest tumor reactivity, immune suppression remains dominant, likely due to regulatory T cells, suppressive myeloid cell populations, and IDO1-mediated immunosuppression [30]. Importantly, the presence of CXCL13^+^ CD8^+^ T cells was correlated with favorable responses to immune checkpoint blockade (ICB) in diverse solid cancers [56]. These observations underscore the need for patient stratification based on T cell abundance and phenotype to optimize the application of immunotherapies. Subsets of patients with BrMs from melanoma or lung cancer have responded positively to ICB, highlighting the potential for immune-based strategies in select patient populations [51].

#### Neutrophil infiltration promotes BrM.

An elevated neutrophil:lymphocyte ratio in the blood is associated with poor prognosis across multiple cancers and has also been recently reported for patients with BrM [57]. Emerging data reveal that neutrophils can infiltrate BrMs, with some degree of variability between different solid cancers [29,58]. Analysis of matched peripheral blood neutrophils and tumor-associated neutrophils (TANs) from patients with BrMs revealed a transcriptionally distinct, proinflammatory phenotype in TANs [29]. While TANs in BrM share some features with those in extracranial tumors, additional inflammatory pathways are induced within the brain, reflecting both disease-type and organ-specific imprinting. Spatial analyses show that TANs localize within perivascular and myeloid cell niches, where they contribute to immune suppression, angiogenesis, and tumor progression, in part through IL-8, G-CSF, and programmed death-ligand 1 (PD-L1)-mediated inhibition of T cells [29]. Mechanistic studies have implicated overexpression of enhancer of zeste homolog 2 (EZH2) in BrM cells as a driver of immunosuppressive neutrophil recruitment via G-CSF production [59]. Targeting this pathway with G-CSF-blocking antibodies or Src inhibitors reduced BrM burden in mouse models, highlighting a potential therapeutic strategy to counter neutrophil-driven immune suppression [59]. Furthermore, preclinical studies demonstrate that neutrophils and TAMs can suppress CD8^+^ T cell function within BrMs, in contrast to the tumoricidal activity observed in matched extracranial tumors [60].

#### TAMs dominate the immune microenvironment of BrMs.

TAMs represent one of the most abundant and diverse immune cell populations in brain malignancies. TAMs exhibit extraordinary diversity, comprising distinct populations that reflect different origins and phenotypes, including resident microglia, MDMs that infiltrate from the peripheral blood, and border-associated macrophages from the leptomeninges [61]. Unlike in primary brain tumors, where microglia are the dominant TAM population, BrMs exhibit a higher proportion of MDMs [28,54]. MDMs play an immunosuppressive role, supporting metastatic outgrowth by secreting factors that enhance angiogenesis, modulate T cell activity, and promote extracellular matrix remodeling. Blocking MDMs through CSF-1R inhibition can disrupt tumor-supportive macrophage functions, although compensatory mechanisms involving perivascular macrophages can blunt therapeutic efficacy [62]. Thus, combining CSF-1R blockade with inhibitors targeting compensatory signaling pathways, such as STAT5, has shown promise in preclinical models for sustained tumor control [62].

#### Factors that influence the immune landscapes of BrMs.

Whole-exome sequencing and WGS, combined with immune cell characterization, identified distinct immune phenotypes specific to genetically defined lung-and breast-derived metastases [63]. In lung-BrMs, TP53 mutations were associated with increased immune cell infiltration and an intriguing dual phenotype: simultaneous activation of cytotoxic CD8^+^ T cells and enrichment of immunosuppressive myeloid populations, including MDMs and microglia with attenuated phagocytic and inflammatory signatures. Similarly, breast BrMs harboring focalized hypermutation (kataegis) exhibited a more inflamed microenvironment, with enhanced T cell infiltration and transcriptional evidence of immune activation across multiple compartments [63]. These findings underscore that, beyond influencing cancer cell state, intrinsic tumor genomics actively sculpt the immune landscape in BrM. They also suggest that specific immunogenomic profiles, such as TP53 mutations or kataegic patterns, could serve as predictors of immune responsiveness, with implications for patient stratification in immunotherapy trials. This convergence of cancer cell plasticity and genetically driven immune landscapes highlights the need for integrated single-cell and immunogenomic approaches to fully resolve the multifaceted biology of BrM. In addition to disease-specific immune alterations, immune cells in BrMs are influenced by non-immune components of the TME, including astrocytes that were discussed earlier. The brain vasculature, in particular, also plays a key role in modulating immune responses, with endothelial and mural cells in the metastatic niche exhibiting an immune-regulatory phenotype, including upregulation of checkpoint molecules such as CD276 [64,65]. Consequently, targeting CD276 with blocking antibodies has shown promise in preclinical models, prolonging survival and enhancing immune infiltration [64], underscoring the potential of vascular-targeted strategies to improve immune access to metastatic lesions.

### Emerging implications of the crosstalk with neurons

Neurons are central for organ-specific functions in health and disease. However, it is taking a surprisingly long time to fully appreciate their role in the pathobiology of BrM, which is currently underrepresented in the field of cancer neuroscience [66].

#### Impact on metastases.

Breast cancer cells that have colonized the brain and formed macrometastases not only can reside near neuronal synapses in a perisynaptic configuration (‘pseudotripartite’) [67] but were also shown to respond to glutamate released from the nearby synaptic cleft via NMDA glutamate receptors. Glutamate uptake by metastatic cells was shown to support tumor cell proliferation [67]. As additional evidence, GABA signaling during early stages of breast-to-brain metastasis can support survival and growth of cancer cells in the brain environment [68]. These diverse neurotransmitters are co-opted by cancer cells to promote their ability to colonize the brain. When and how this neural adaptation of metastatic cells occurs has begun to be elucidated. In a neuronal-dependent manner, breast cancer cells activate SRRM4, a splicing factor specifically required for neural cell differentiation [69]. SRRM4 induces alternative splicing of REST, reducing its repressive activity and leading to the activation of a gene program that promotes tumor growth, synaptic signaling, and stress resistance [69]. By contrast, SCLC hijacks synaptic signaling to promote tumor growth already at the primary site, a process that persists during metastatic spread to the brain, where GABAergic and glutamatergic synaptic inputs provide a proliferative advantage [70,71].

#### Impact on the brain.

An understudied problem in the field is the impact of BrM on neurocognitive functions. Patients with newly diagnosed BrMs that possess minimal functional impairment often exhibit cognitive dysfunction [72]. Recently, it was demonstrated that cancer cells from distinct primary cancer types that harbor distinct genetic profiles can differentially influence the function of neural circuits in the brain [73]. This study suggests that elucidating underlying molecular signatures, coupled with electrophysiology approaches, may provide a roadmap to better understand and treat neurocognitive defects in patients with metastases. Importantly, this work demonstrates that experimental models could be used to study the impact of metastases on brain activity. Beyond the limited experimental work dedicated to understanding the impact on brain function, a major limitation is also the lack of a standardized neurocognitive evaluation of patients with BrM as part of the standard of care. Thus, the clinical implementation of tools such as the Amsterdam Cognition Scan (ACS) [74], which allows individuals to conduct a self-administered, online cognitive test, would contribute important data to the multidimensional analyses of patients and associated tissue samples. A comprehensive approach integrating molecular and imaging profiles, liquid biopsies, clinical history, and neurocognitive assessments is greatly needed to fully characterize the impact of BrM and improve patient care (Box 2).

## Metabolism

Disseminated tumor cells undergo profound metabolic reprogramming following colonization of the brain. These adaptations alter how BrM cells take up and utilize key metabolites while also reshaping the microenvironment through the release of metabolic byproducts [31,75–78]. While some metabolic pathways remain consistent among BrM cells from different cancer types, distinct metabolic processes are also evident, reflecting metabolic heterogeneity. Moreover, BrMs may exhibit metabolic plasticity by optimizing the use of the same key nutrients more efficiently than the primary tumor. Alternatively, they can exhibit metabolic flexibility, harnessing available nutrients in the brain microenvironment to meet their energy demands, biosynthetic needs, and redox balance. Importantly, these altered, adaptive metabolic states are observed even in BrM cells with similar oncogenomic profiles, underscoring their metabolic heterogeneity and ability to dynamically adjust to environmental pressures. This suggests that, when confronted with adverse, non-permissive microenvironments, tumor cells can leverage multiple survival mechanisms. We discuss in the following section the main findings related to the metabolism of BrMs.

### Core metabolic pathways fuel the formation of BrMs

The metabolic adaptations in tumor cells are influenced by their oncogenomic state, secretome, metastatic fitness, and interactions with the local microenvironment. Increased glycolytic and pentose phosphate pathway activity, driven by oncogenic mutations [e.g., c-MYC, human EGFR 2 (HER2), PIK3CA], enhances NADPH production and nucleotide synthesis in breast BrM [79]. Enhanced glucose utilization, driven by the upregulation of glycolytic enzymes and transporters, supports energy production, contributes to microenvironment acidification, and promotes therapy resistance and metastatic outgrowth in breast and lung BrMs [80–83]. Notably, a metabolic shift from oxidative phosphorylation (OXPHOS) to glycolysis is observed in both primary brain tumors and NSCLC BrMs [83,84]. By contrast, BrMs arising from TNBC exhibit dual enrichment in glycolysis and OXPHOS, enabling both rapid, oxygen-independent energy generation and efficient, oxygen-dependent ATP production [85].

Altered mitochondrial biogenesis and dynamics result in OXPHOS modulation, which is critical in maintaining cellular energy needs, redox balance, and biosynthetic pathways [86–92]. BrMs frequently upregulate the production of mitochondrial proteins such as ALDH9A1, FH, and COX7B, or employ the GABA shunt to drive this oxidative shift [24,85,93–98]. Additionally, elevated acetate metabolism, mediated by ACSS2, provides an alternative energy source for BrMs, enabling survival under metabolic stress [99,100]. Recent studies have further revealed distinct metabolic profiles between latent and aggressive tumor cells, with latent cells prioritizing oxidative glutamine metabolism, lipid catabolism, and reactive oxygen species (ROS) mitigation to maintain metabolic stability [101–105], whereas aggressive cells amplify glucose metabolism, glycolysis, and fatty acid synthesis to fuel rapid proliferation [101,102,106,107].

### Amino acids as alternative nutrient sources for BrMs

To survive in the nutrient-scarce brain microenvironment, metastatic cells also rely on enhanced amino acid transport and catabolism. Elevated expression of amino acid transporters, such as LAT1, facilitates the uptake of essential amino acids including branched-chain amino acids (BCAAs), phenylalanine, methionine, tryptophan, and tyrosine across the BBB [108,109]. Increased BCAT1 expression and BCAA catabolism reduces α-ketoglutarate levels and inhibits the m6A demethylase ALKBH5, which correlates with poor prognosis in NSCLC [110]. Similarly, TNBC BrMs depend on glutamine and BCAA catabolism to sustain gluconeogenesis under glucose-limited conditions [111].

Beyond direct nutrient acquisition, BrM cells actively reshape the metabolic landscape of the brain microenvironment. For example, breast BrMs can release extracellular vesicles carrying miR-199b-5p, which downregulates solute carrier expression in both astrocytes (SLC1A2/EAAT2) and neurons (SLC38A2/SNAT2 and SLC16A7/MCT2) [112]. As a result, the metabolic coupling between astrocytes and neurons is disrupted, leading to the accumulation of glutamine, glutamate, and lactate in the extracellular microenvironment that can be taken up by the metastatic cells to promote their growth [112]. Coupling between BrM cells and astrocytes can further promote signaling in cancer cells in an amino acid-dependent manner. For example, in lung BrMs, astrocyte-derived Wnt5 induces the expression of metabotropic glutamate receptor 1 (mGluR1) [113]. High levels of mGluR1 stabilizes EGFR in the cancer cells, in a glutamate-dependent manner, leading to activation of downstream ERK signaling and increased cancer cell proliferation [113]. Elevated asparagine levels, driven by upregulation of asparagine synthetase (ASNS), are also associated with BrM and poor outcomes [114]. Furthermore, MYC overexpression enhances amino acid metabolism by upregulating serine, asparagine, and proline biosynthesis pathways, collectively supporting BrM growth [79].

Melanoma, breast, and clear cell renal cell carcinoma (ccRCC) BrMs frequently upregulate phosphoglycerate dehydrogenase (PHGDH), the rate-limiting enzyme in the serine biosynthetic pathway. This metabolic adaptation compensates for serine deficiency in cerebral tissues, enabling metastatic cells to maintain nucleotide biosynthesis and redox balance [115]. Additionally, glial cells in the brain facilitate metabolic adaptations by promoting the generation of valine intermediates and products essential for gluconeogenic valine catabolism. This pathway is markedly enhanced in metastatic cells through the upregulation of HIBCH, a key enzyme in valine metabolism [116]. Together, these studies reveal the importance of amino acid uptake or synthesis by cancer cells that have colonized the brain.

### Lipid metabolism in BrMs

During BrM, lipid metabolism is also reprogrammed, enhancing lipid synthesis, uptake, and fatty acid oxidation. These changes not only support microenvironment interactions, membrane remodeling, and energy production under stress, but also aid immune evasion and therapy resistance [117–121]. For instance, in HER2^+^ breast cancer, ^13^**C-glucose labeling studies** reveal that glucose-derived palmitate is a key contributor to fatty acid synthesis, promoting metastatic proliferation [107,122]. Overexpression of **SREBP1**, a master regulator of lipid synthesis, further enhances fatty acid metabolism, enabling tumor adaptation to the TME [123]. In line with these observations, **RARRES2**, a negative regulator of lipid metabolism genes such as FASN, SCD, HMGCR, CPT1A, and PTDSS1, is significantly decreased in patients with BrM [124,125]. Increased expression of LPCAT1 and FABP7 in NSCLC and breast BrMs correlates with poor prognosis [126,127].

### Metabolic influence of the crosstalk with the BrM microenvironment

The metabolic alterations in cancer cells also play a critical role in shaping their fitness and interactions within the TME. Additionally, brain-resident cells dynamically shape the metabolic landscape and behavior of metastatic cancer cells. This bidirectional interaction induces metabolic adaptations in both cell types, facilitating nutrient acquisition, immune evasion, and adaptation of metastatic cells to the brain microenvironment. In addition to the studies above related to the metabolism of amino acids, BrMs engage astrocytes through PCDH7-**Cx43 gap junctions**, transferring **cGAMP** to activate the **cGAS–STING pathway**, which induces cytokine release and tumor proliferation [128]. BrMs also reprogram brain-resident cells, such as astrocytes and microglia, to supply fatty acids and promote redox balance. Moreover, BrMs frequently adopt a neuronal-like metabolism, including by adopting a GABAergic phenotype and upregulating glutamate signaling pathways [67,98,129], further enhancing their ability to thrive in the CNS.

Overall, recent clinical and preclinical studies have revealed the heterogeneous metabolic landscape of BrMs and identified actionable vulnerabilities. Future investigations aimed at targeting these dependencies could open new therapeutic avenues and improve survival outcomes.

## Experimental models and technological advances

### Revisiting experimental models of BrM

The quality and the translational value of preclinical research relies on how accurately experimental models reproduce the human disease. Despite over two decades of research, there remains a lack of experimental models that fully recapitulate the entire metastatic cascade from primary tumor formation to the establishment of macrometastases in the brain. However, advances in genetically engineered mouse models (GEMMs) have allowed the study of spontaneous BrMs [130,131]. Additionally, metastatic cell lines with the ability to colonize the brain from an orthotopic inoculation have also been established [132–136].

**Figure 3.**
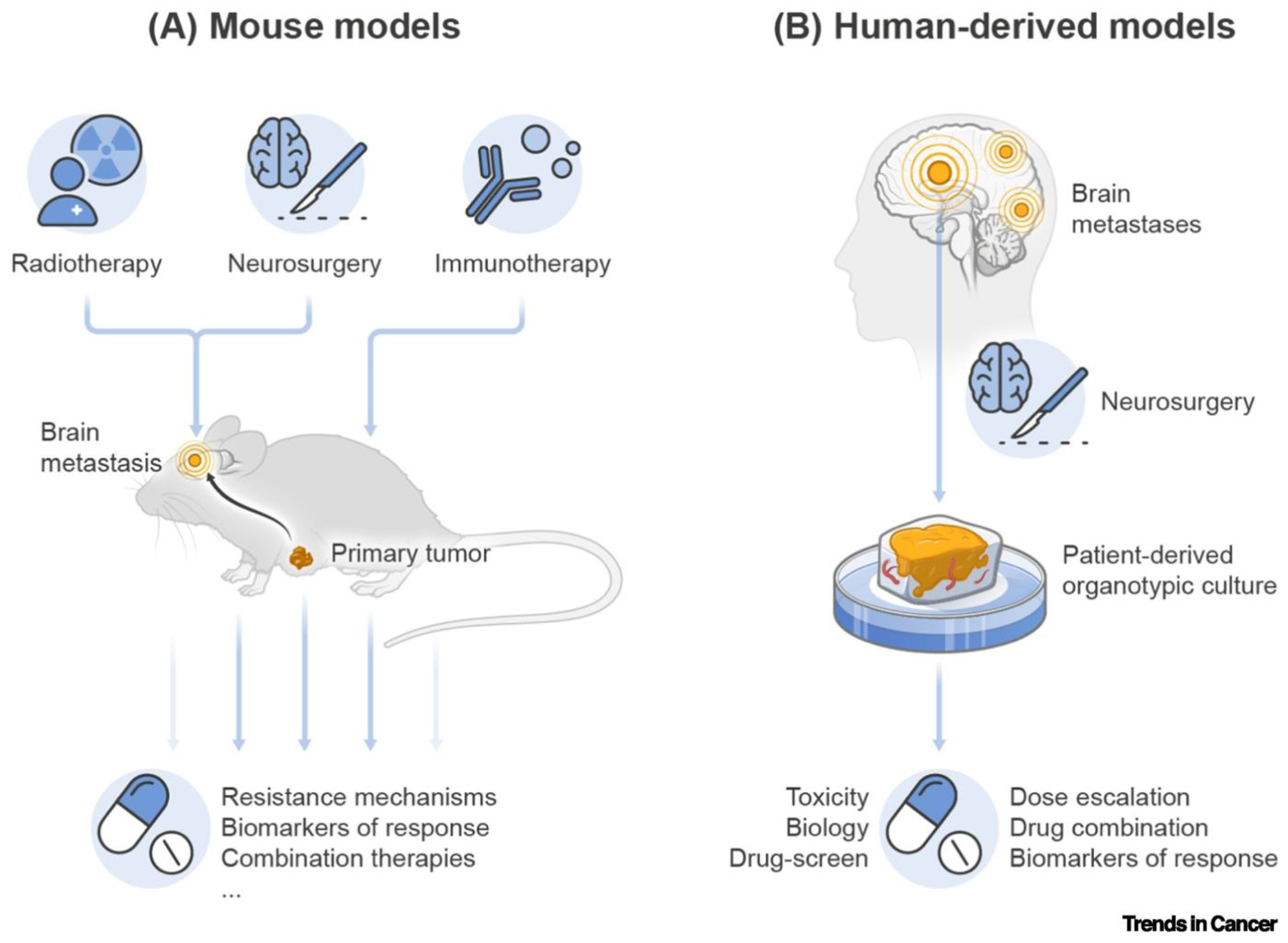
Advanced experimental models for brain metastasis research. (A) The lack of experimental models that form spontaneous metastases from orthotopic injections and/or from genetically engineered mouse models that develop primary tumors remains a barrier to progress in the field. However, more sophisticated models that have incorporated local (radiotherapy, neurosurgery) and systemic (i.e., immunotherapy with immune checkpoint blockade antibodies) therapies have now been developed. The applications of these therapeutic approaches permit the study of how these interventions impact biology as well as the identification of molecular mechanisms that might be responsible for resistance. In addition, such models may reveal biomarkers that might identify alternative combinations that might be more effective, which could avert resistance. (B) The validation of patient-derived organotypic cultures obtained from fresh neurosurgeries is an emerging strategy to access human brain metastases. Such approaches may elevate research with these models beyond the descriptive nature of human-based research, enabling functional experiments that could reveal drug sensitivity of certain patients to a specific drug, for instance.

Despite these advances, the most commonly used approach remains the inoculation of organotropic metastatic cell lines into the systemic circulation [137]. For an in-depth review of these methodologies, we refer the reader to previous publications [137]. In recent years, the availability of syngeneic organotropic cell lines has significantly expanded [137]. In addition, preclinical models have begun incorporating therapeutic approaches that are part of the standard of care, including radiotherapy [138], neurosurgery [139], and immunotherapy [47] (Figure 3A). While existing models still fall somewhat short of fully reproducing the diverse clinical presentations of BrM – including synchronous vs metachronous, single vs oligometastatic vs multiple, and brain-only vs multiorgan dissemination – the field is evolving towards more sophisticated models by incorporating these critical aspects.

### Patient-derived organotypic cultures in BrMs

In addition to *in vivo* models, experimental *ex vivo* approaches allow direct testing of therapies on human BrM samples. The use of patient-derived organotypic cultures (PDOCs) [140] provides a clinically relevant platform to evaluate therapies across multiple patients before initiating clinical trials [140]. This *ex vivo* methodology has demonstrated the ability to predict patient responses, assess local toxicity, identify biomarkers of drug response, test combination therapies, and even be escalated into medium-throughput drug screening platforms [139] (Figure 3B). Overall, PDOCs are emerging as a human *ex vivo* approach that has the potential to optimize the development and success of clinical trials in oncology.

### Modeling standard-of-care therapies for BrMs

#### Neurosurgery.

Approximately 40% of patients with BrM undergo neurosurgical resection [141]. Surgery is typically reserved for patients with one or a limited number of accessible metastases that involve local neurological compromise while the extracranial disease and the general status of the patient remain stable. Preclinical models of experimental neurosurgery, applied to an intracranially implanted NSCLC brain-tropic cell line, similarly recapitulated the prolonged survival [139]. However, all mice eventually developed local recurrence and succumbed to the disease [139], mirroring clinical data where 60% of patients experience local relapse within 1 year of surgery [141]. This model thus provides a useful tool to study post-surgical recurrence, which remains a major clinical challenge without an established standard-of-care treatment plan.

Recent data indicate that vascular co-option plays a critical role in local relapse, particularly at the invasive fronts surrounding the metastasis [139]. The clinical relevance of this process is supported by patient data [39,142,143]. Thus, targeting the vascular co-opting tumor cells at the invasive front represents a potential adjuvant strategy to avoid local relapse after surgery.

#### Radiotherapy.

Radiotherapy is another cornerstone of BrM treatment. Historically, whole-brain radiotherapy (WBRT) was the standard approach, but its limited clinical benefits [144] have led to a shift towards focal irradiation approaches such as stereotactic radiosurgery (SRS) and stereotactic fractionated radiation (SFR). These targeted approaches provide improved local control [144], although challenges remain for large metastases and the risk of radiation necrosis persists [144].

Preclinical models incorporating WBRT have reported an acquired mechanism of radioresistance induced by the brain microenvironment [138]. Molecular analyses revealed that blocking the resistance pathways through RAGE inhibitors can improve responses in mice, leading to an ongoing clinical trial (NCT05635734). Additionally, liquid biopsy detection of S100A9 is now being evaluated in a prospective clinical study as a potential biomarker of radiotherapy resistance [138,145].

More recently, preclinical modeling of SRS in breast-to-brain metastasis revealed that while CD8^+^ T cells infiltrate BrMs, they fail to enhance radiotherapy efficacy due to local immune suppression [60]. This was in contrast to a robust CD8^+^ T cell response for the same tumor but growing in an extracranial site (orthotopic mammary tumor). Neutrophils and macrophages were identified as likely mediators of radioresistance, supporting the integration of targeted myeloid reprogramming strategies to improve the efficacy of radiotherapy in BrM.

#### Immunotherapy.

The immune-suppressive TME also poses a major challenge to ICB in BrMs [47]. Despite CD8^+^ T cell infiltration, these cells remain functionally impaired, failing to mount an effective antitumor response even when ICB is administered [47,60]. Studies in preclinical models have revealed multiple resistance mechanisms in the TME, as discussed earlier. For instance, targeting signaling pathways such as STAT3 or CD276, or reprogramming myeloid cells, may help to reverse immune suppression and restore T cell activity. Notably, clinical trials are underway testing STAT3 inhibitors like silibinin (NCT05689619), informed by mechanistic work demonstrating paracrine suppression of T cells by reactive astrocytes [47]. Moving forward, integrating immune-modulating therapies with ICB – and evaluating these in preclinical systems that replicate the full complexity of the BrM microenvironment – will be critical in overcoming current barriers to immunotherapy efficacy in the CNS.

### Technological advances in studying BrM

#### Clinical imaging modalities.

Advanced imaging technologies play a crucial role in understanding BrM biology and progression, as well as in diagnosis, treatment planning, and disease monitoring. Magnetic resonance imaging (MRI) remains the gold standard, offering multiple specialized sequences that provide complementary insights into tumor characteristics and the surrounding microenvironment [146–148]. T1-weighted imaging with gadolinium enhancement highlights disruption of the BBB, characteristic of most BrMs, typically appearing as ring-enhancing lesions [146]. T2-weighted and FLAIR sequences are crucial for detecting associated edema and non-enhancing tumor components, helping to define the extent of disease [146]. Diffusion-weighted imaging and apparent diffusion coefficient maps help to distinguish metastases from other intracranial lesions and to assess treatment response [149]. Susceptibility-weighted imaging provides high sensitivity for the detection of microhemorrhages and calcifications [150]. Functional imaging techniques such as MR spectroscopy provide metabolic information to distinguish metastases from primary brain tumors or radiation necrosis [151], while perfusion-weighted imaging evaluates tumor vascularity and can help to predict treatment response [152].

Beyond MRI, positron emission tomography (PET), particularly when combined with computed tomography (CT) or MRI, enhances imaging capabilities by integrating metabolic information. This has proved useful in distinguishing viable tumor from treatment-related changes [153]. Different radiotracers can provide specific information. For example, FDG-PET remains widely used to identify metabolically active lesions [153], while amino acid tracers such as FET or FDOPA improve tumor detection [153]. Here, novel criteria allowing metabolic response assessment of BrMs using amino acid PET have been developed (PET RANO BM 1.0) and should facilitate the development of novel therapies for patients with BrMs by introducing innovative endpoint clinical trials [154]. Novel radiotracers targeting specific molecular features of different cancers continue to expand the utility of PET in the personalized assessment of BrM [153].

#### Microscopy technologies in BrM research.

Microscopy-based approaches have provided high-resolution insights into the cellular and molecular interactions shaping the metastatic niche. Electron microscopy reveals the ultrastructural details of cellular interactions in the BrM microenvironment, displaying the precise details of synaptic connections and vascular interfaces [35]. Immunohistochemistry and immunofluorescence staining in combination with high-end super-resolution light microscopy continue to be essential for protein-level analysis and spatial distribution of biomarkers, allowing identification of specific cell populations and their states within the metastatic niche [35,67,155]. Innovative imaging technologies are enabling real-time visualization of metastatic processes *in vivo*. Intravital imaging allows direct visualization of tumor and immune cell behavior, vascular interactions, and therapeutic responses in living organisms [156]. Additionally, calcium imaging – traditionally used in neuroscience – allows functional analysis of neural activity in metastatic niches, providing insights into how tumor cells might integrate into neuronal circuits [157].

#### Emerging neuroscience-derived technologies.

Advances in neuroscience tools and functional imaging are now being leverage to study BrMs with unprecedented resolution. Dyes originally developed for neuroscience research, such as Sulforhodamine 101 (SR101), traditionally used to label astrocytes, can be adapted to study astrocyte–tumor cell interactions and network formation [158]. Similarly, neural network tracing technologies, including viral tracers and molecular tools, have the potential to map the cellular networks formed by metastatic cells and their interactions with the brain microenvironment [159]. Trans-synaptic tracers, such as modified rabies viruses, could be adapted to determine the extent and specificity of tumor cell integration into neural circuits, an approach previously used to study primary brain tumors [160].

Beyond structural mapping, optogenetic and chemogenetic technologies provide tools for functional interrogation of tumor–microenvironment interactions. Optogenetic approaches, which use light-sensitive proteins to control cellular activity, could be harnessed to selectively manipulate specific populations of tumor cells or surrounding microenvironmental partners with unprecedented temporal and spatial precision [161]. Similarly, chemogenetic tools like designer receptors exclusively activated by designer drugs (DREADDs) allow selective modulation of signaling pathways within metastatic cells or supporting cells in the brain microenvironment [162].

## Therapeutic advances

With advances in systemic therapies for many cancers, BrMs are becoming increasingly treatable with a range of medical approaches. Breakthroughs in target identification, drug design, and clinical trial design have led to improved outcomes, including prolonged survival for patients with BrMs. Such advances raise the possibility that, in some cases, disease control may be achieved without local therapies such as surgery or radiation. However, the CNS remains a uniquely challenging disease site, in part due to the BBB and BTB (in macrometastases), which impose significant constraints on drug penetration. These barriers comprise specialized multicellular layers, including endothelial and mural cells, with tight junctions and adherens junctions that regulate CNS entry as well as intricately regulated efflux pumps, which clear the CNS of toxins, drugs, and other molecules. As a result, in some cancers with a high burden of BrMs, CNS progression often becomes the primary determinant of survival and quality of life [163].

In recent years, clinical trials have increasingly included patients with BrMs and, in some cases, with CNS-specific endpoints, allowing a more refined evaluation of emerging agents and their efficacy in the CNS. These developments are leading to a growing number of effective treatments, including targeted therapies, antibody–drug conjugates (ADCs), and ICB.

### Small molecule inhibitors

Targeted therapies, including small molecule tyrosine kinase inhibitors (TKIs), have significantly improved both overall survival (OS) and CNS-specific outcomes for a growing number of patients with actionable genomic alterations. One of the most notable examples is the successive generations of TKIs targeting the EGFR, which have become highly effective in the 10–15% of NSCLCs that harbor activating mutations in the EGFR – including exon 19 deletions, exon 21 substitutions (including L858R), and the exon 20 resistance mutation T790M. The third-generation EGFR-TKI osimertinib, which has demonstratable enhanced BBB penetration in preclinical models compared with earlier EGFR-TKIs, has shown significant clinical benefits in patients with BrM [164]. A pivotal study showed that osimertinib extended progression-free survival (PFS) to 15.2 months, compared with 9.6 months with prior-generation TKIs (gefitinib/erlotinib; HR = 0.47, *P* < 0.001). Moreover, in the overall study population – including patients without known BrMs – CNS progression was reduced from 15% to 6%, underscoring its superior intracranial efficacy [165,166]. A similar trend has been observed with inhibitors targeting anaplastic lymphoma kinase (ALK), which are relevant in approximately 5% of NSCLC cases. The second-generation AKL-TKI alectinib significantly reduced the risk of CNS progression compared with the previous-generation crizotinib (12% vs 45%; HR = 0.16, *P* < 0.001) [167]. A third-generation TKI, lorlatinib, targeting rearrangements in ALK and ROS1, which is highly brain penetrant, demonstrated a 46% intracranial response rate in a heavily pretreated NSCLC population with ALK rearrangements (72% with baseline BrM and 52% with two or more prior TKIs). Furthermore, lorlatinib markedly reduced the 12-month incidence of CNS progression compared with crizotinib, both in patients with baseline BrM (7% vs 72%) and in those without (1% vs 18%) [168,169].

The successful small-molecule targeting of KRAS alterations, which occur in up to 30% of all NSCLC cases (as well as in other malignancies), has recently been demonstrated, with emerging data supporting CNS efficacy. Sotorasib, a KRAS G12C inhibitor, demonstrated a trend towards prolonged intracranial PFS in patients with baseline BrM compared with docetaxel chemotherapy (9.6 vs 5.4 months; *P* = 0.37). In a cohort of patients with untreated BrM, the KRAS G12C inhibitor adagrasib demonstrated an intracranial objective response rate (ORR) of 42%, a disease control rate of 90%, and median OS of 11.4 months [170,171].

In rarer genomic lesions, such as ROS1 and TRK fusions, targeted therapies have also shown notable intracranial efficacy. The ROS1/TRK inhibitor entrectinib demonstrated an 80% intracranial ORR, a median intracranial duration of response (DoR) of 12.9 months, and a median intracranial PFS of 8.8 months in ROS1 fusion-positive, TKI-naïve NSCLC patients with baseline CNS metastases [172]. Similarly, in a comparable patient population, repotrectinib showed a 88% intracranial response rate [173].

Beyond lung BrM, small molecule inhibitors targeting the MAPK pathway have also improved CNS outcomes, particularly in the 40–50% of melanoma patients with mutations in BRAF, as well as in smaller proportions of patients with other primary cancers. Specifically, combinatorial inhibition of BRAF and MEK with dabrafenib and trametinib demonstrated an intracranial response of 58% in patients with BRAF-V600E asymptomatic and previously untreated melanoma BrMs [174]. In a heavily pretreated population of patients with BRAF-mutant melanoma and BrM (88% with prior local therapy and 88% with prior BRAF/MEK inhibition), the combination of encorafenib and binimetinib led to a 33% intracranial response rate [175].

In HER2-expressing malignancies, the most prevalent of which are breast cancers, multiple TKIs have been investigated for CNS activity. One of the most well-studied examples is tucatinib, which, in combination with trastuzumab and capecitabine, resulted in a 68% reduction in intracranial progression or death versus placebo, and a CNS PFS extension to 9.9 months vs 4.2 months [176].

### ADCs

ADCs have emerged as a promising class of potent agents for BrM, particularly in HER2^+^ breast cancer. Trastuzumab deruxtecan (T-DXd) has demonstrated substantial intracranial activity, with prospective trials such as DEBBRAH, TUXEDO-1, and DESTINY-Breast12 [177–181] reporting objective intracranial responses of approximately 60–70%. Consequently, T-DXd is now a recommended treatment for HER2^+^ breast BrM, according to contemporary guidelines [182,183].

Beyond HER2-targeted ADCs, several other ADCs are currently under development or clinical evaluation, targeting alternative oncogenic drivers. Two promising targets include human EGFR 3 (HER3) [184], another member of the EGFR family that plays a role in tumor progression and therapy resistance, which has been shown to be frequently overexpressed in BrMs of NSCLC and breast cancer [185], and trophoblast cell surface antigen 2 (TROP2), a transmembrane glycoprotein overexpressed in various epithelial cancers, including breast and lung cancer.

For these and other ADC targets to be effective against CNS metastases, several pharmacokinetic and physicochemical factors must be optimized, including brain penetration, lipophilicity, linker technology and stability, and the drug:antibody ratio [186]. Ongoing clinical trials are evaluating HER2-, HER3-, and TROP2-targeting ADCs to determine their efficacy in patients with breast, lung, and other cancers.

### Immunotherapies for CNS metastases

The advent of ICB has transformed cancer therapeutics over the past two decades. Specifically, the ability of ICB to induce durable responses, in some cases leading to cures in the metastatic disease setting, has had a profound impact on cancer therapeutics and drug development in general. However, in keeping with the traditional paradigm of drug development, patients with CNS metastases were excluded from the early trials of ipilimumab (anti-CTLA4 antibody) as well as pembrolizumab and nivolumab (anti-PD1 antibodies), and their combinations.

Nonetheless, initial observational data from metastatic melanoma patients treated off protocol, or on the early-access programs to ipilimumab, showed potential activity in CNS metastases. This was later confirmed in a prospective Cytokine Working Group trial [187,188], which demonstrated that ipilimumab exhibited equivalent activity in parenchymal BrMs and extracranial disease, provided that patients were not requiring corticosteroids at the time of ICB initiation. Specifically, ipilimumab produced durable intracranial responses in approximately 15% of asymptomatic patients who were not receiving steroids. Subsequent trials in metastatic melanoma specifically enrolled patients with BrMs in treatment arms receiving single-agent anti-PD1 antibodies, with both pembrolizumab and nivolumab achieving intracranial ORRs for approximately 22% of patients [189,190]. While these results were encouraging, it still fell short of the typical 35–40% ORR consistently observed for ICB in extracranial melanoma.

The combination of anti-CTLA4 and anti-PD1 therapy was subsequently evaluated in a large Phase 3 trial, which compared this dual ICB to single-agent anti-CTLA4 therapy. The study suggested a small advantage of dual therapy over single-agent anti-PD1, albeit at the cost of increased toxicity. However, two independent Phase 2 trials in patients with melanoma BrM confirmed that dual ICB therapy exhibits excellent intracranial activity, with response rates at least equivalent to extracranial disease, producing durable intracranial ORRs in more than 50% of patients [191,192]. This resulted in the establishment of dual ICB as a standard-of-care regimen in this specific patient population.

Further analysis of these trials demonstrated that patients requiring steroids at the time of ICB initiation derived significantly less benefit, igniting interest in approaches to reduce or eliminate steroid use before initiating dual ICB [193]. Strategies to achieve this include surgical resection, radiosurgery, or the early use of BRAF-targeted therapy in BRAF-mutant melanoma [194,195]. Combination therapies, such as nivolumab plus dabrafenib/trametinib, have also demonstrated CNS activity in BRAF-mutant melanoma, with intracranial responses observed in four of seven evaluable patients, including two with complete responses [196]. Similarly, combination PD-L1 inhibition with atezolizumab plus BRAF/MEK inhibition (with vemurafenib/cobimetinib) achieved an intracranial ORR of 42% in BRAF-V600-mutant cases, although grade ≥3 toxicity was high (68%) [195]. These findings have also encouraged the inclusion of patients with asymptomatic CNS metastases in an increasing number of clinical trials for systemic immunotherapy agents. For example, an ongoing trial is evaluating the role of the anti-lymphocyte activation gene 3 (LAG3) antibody relatlimab in combination with nivolumab for melanoma BrM (NCT05704647).

Beyond melanoma, ICB has also been investigated in patients with NSCLC BrM, yielding promising results. A single-arm trial demonstrated an intracranial ORR of 22% for pembrolizumab in asymptomatic patients [189]. Meanwhile, the addition of the anti-PD-L1 antibody atezolizumab to chemotherapy resulted in a intracranial ORR of 42.5%, even in a population where more than half of the patients were receiving steroids [197]. Additionally, a Japanese study reported an intracranial ORR of 50% for ipilimumab and nivolumab when combined with chemotherapy in NSCLC patients with previously untreated BrMs [198]. Pembrolizumab has also been evaluated in BrMs from several tumor types, where it achieved a clinical benefit rate of 42% [199]. By contrast, breast BrMs and SCLC BrMs have not shown evident clinical responses to ICB strategies to date, as reported for certain melanoma and NSCLC BrM patients, underscoring the need to more deeply understand and overcome mechanisms of immune suppression.

### Future directions to bridge key clinical gaps

In addition to the continued development of increasingly safe and effective therapies for cancer writ large, several key areas of investigation are necessary to optimize the integration of medical therapy into the treatment of BrM, which has historically relied on local treatment modalities such as radiotherapy and surgery. A primary challenge lies in understanding the interactions and order of deployment for multimodal treatments. This is particularly relevant because local therapies remain quite effective and are frequently used in both the upfront and salvage settings, especially as BrMs are often detected when they are large and/or symptomatic due to the absence of routine CNS screening in many solid tumor surveillance paradigms [200–202]. At these larger sizes, medical therapies face inherent limitations and are rarely durable in the CNS. One notable example is the heightened risk of potentially morbid radionecrosis in patients treated with both immunotherapy or ADCs in combination with radiotherapy [203–205]. Additionally, a critical gap remains in understanding the impact of corticosteroids – widely used to manage cerebral edema – on immunotherapy efficacy, as some evidence suggests reduced treatment benefit, though this finding cannot be generalized [206]. Finally, with some exceptions, there remains a lack of clear data on the role of systemic therapies in patients with CNS-restricted cancer progression, a growing yet understudied subset of the metastatic population. Given the complex interactions of cancer-directed and palliative treatments, and of patient neurological condition and goals, there is an increasing recognition that specialized multidisciplinary care teams may be best for clinical decision-making and care coordination [207,208].

Another critical challenge lies in deciphering the distinct biological mechanisms that drive resistance in the CNS compared with extracranial disease. A deeper understanding of these mechanisms could help to refine therapeutic strategies and identify new vulnerabilities specific to BrM. One promising avenue is the potential role of CNS sampling to track molecular evolution and guide treatment decisions [209]. For example, discordance in HER2 expression has been observed in more than 50% of patients with initially HER2-null primary breast cancer, where the sampled BrMs were found to have transformed into HER2-low-expressing tumors. Additionally, the constitutively active p95 isoform of HER2 has been shown to be more prevalent in BrMs than in matched primary tumors [210,211], raising important questions about intrapatient tumor evolution and treatment implications. Similar studies have identified substantial variability in EGFR mutations, PD1 expression levels, and other potentially actionable biomarkers between primary and metastatic lesions [6,212]. These findings highlight the need for further investigation into the molecular and genetic heterogeneity of BrMs, which may ultimately inform more precise therapeutic strategies tailored to the CNS microenvironment.

Additionally, there are multiple unanswered questions in the immunotherapy field, requiring further exploration. A major area of debate is the precise nature of the immune response within the CNS and whether meaningful T cell activation occurs intracranially. Additionally, the optimal integration of ICB with SRS remains unclear, including determining the best strategies for combining these modalities and designing randomized trials to assess their efficacy. The role of the TME in shaping ICB responses is also a critical unknown, including whether immune-modulating therapies must be brain penetrant to be effective. Further studies are needed to clarify the best methodology to assess intracranial response, as current criteria such as RECIST, modified RECIST, and RANO-BM may not fully capture CNS-specific treatment effects. To this end, the novel PET RANO-BM 1.0 guidelines have been developed for metabolic response assessment of BrMs using amino acid PET [154]. Another ongoing debate is how to define symptomatic versus asymptomatic BrMs and whether these classifications hold prognostic or predictive value for treatment response. Moreover, given the evidence that corticosteroids negatively impact ICB efficacy, identifying steroid-sparing approaches remains an urgent priority. Finally, for patients with leptomeningeal disease (Box 3), a particularly aggressive manifestation of CNS involvement, the potential of intrathecal ICB delivery to circumvent the limitations of systemic immunotherapy remains an open and important question.

Despite the progress in systemic therapies, clinical trials investigating CNS-specific activity remain both rare and often limited in scope. Historically, hesitancy in including patients with BrM has largely stemmed from concerns over poor survival outcomes, and the confounding influence of prior local therapies. However, with the increasing recognition of BrM as a distinct and treatable entity, there is a growing need for trials that specifically evaluate therapeutic efficacy in the CNS. When appropriately designed, these studies require standardization of outcome measures, including the routine incorporation of CNS-specific endpoints, such as intracranial ORR and CNS PFS, rather than relying solely on overall PFS/OS. Additionally, while most trials focus on treating established metastases, a key priority is the development of ‘secondary CNS prevention’ strategies aimed at delaying or preventing CNS progression in high-risk patients, including those with a high burden of systemic disease. Achieving this goal will require adequately powered studies, as well as significant financial investment to support these trials. A promising approach to address these challenges is the use of window-of-opportunity studies, which allow early evaluation of drug activity within the CNS while minimizing accrual and procedural barriers. Expanding the scope of clinical trials to incorporate these strategies will be essential in advancing the next generation of therapies for BrM.

Ultimately, the future of BrM treatment lies in the convergence of systemic and local approaches, underpinned by a deeper biological understanding of the CNS TME. Continued innovation in trial design, biomarker discovery, and immune-based therapies will be key to transforming BrM from a historically intractable condition to one that is increasingly manageable, if not curable, for a growing number of patients.

## Prevention

Despite important advances in the treatment of CNS metastases, the current strategies remain largely reactive, with limited ability to alter the natural history of brain-metastatic disease in most patients. Improved survival rates have been modest for most patients, aggressive local treatments are still required, and brain relapse frequently occurs even among patients who initially respond well to systemic therapies such as immune checkpoint inhibitors. These limitations underscore the urgent need to develop effective prevention strategies, which may offer a more sustainable approach to improving survival outcomes [7]. Encouragingly, significant efforts in recent years are moving prevention from an aspiration to a real possibility.

### Identification of patients at high-risk of developing CNS metastases

**Figure 4.**
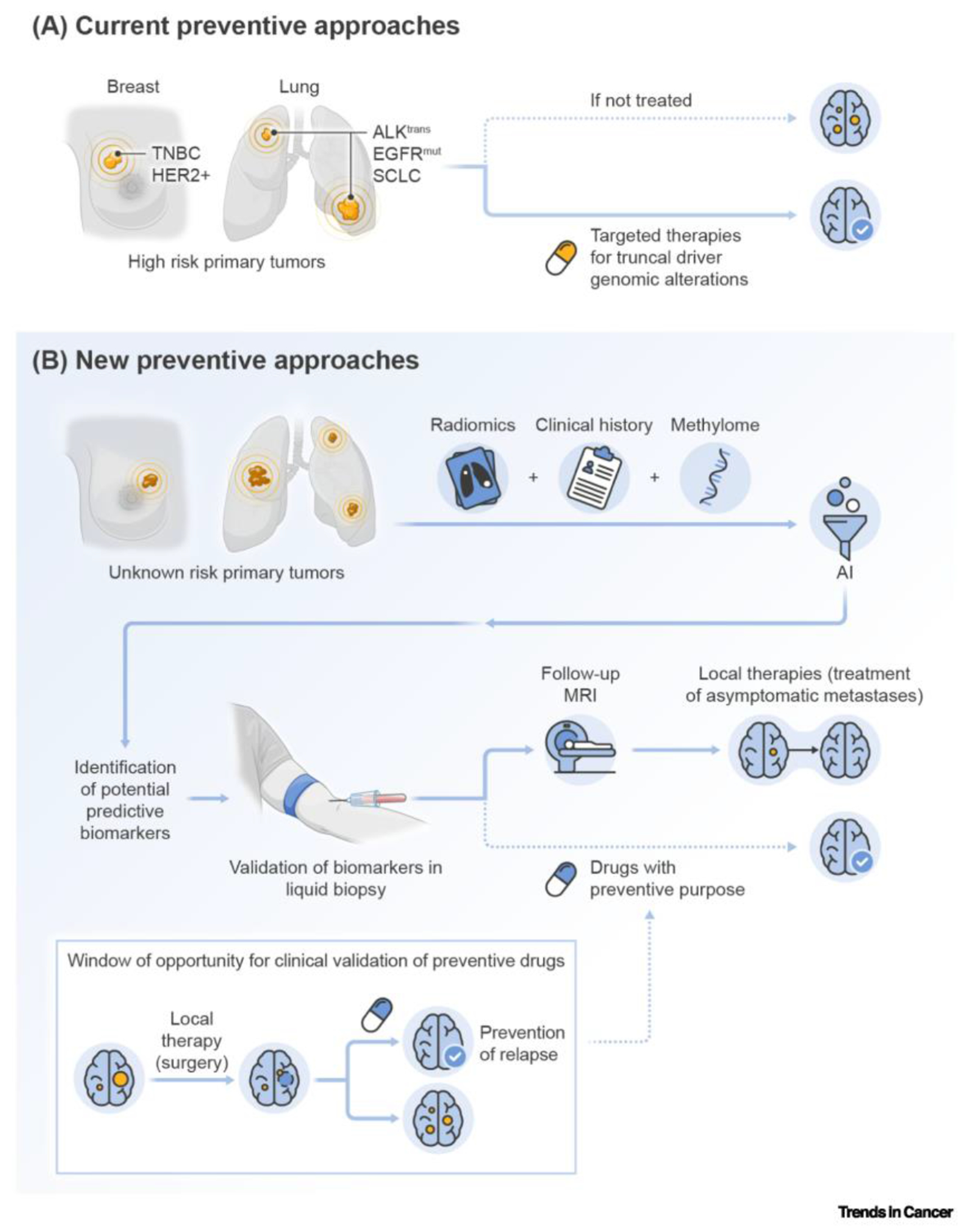
Preventive approaches for brain metastases. (A) It is possible to identify primary tumors linked to specific molecular alterations that exhibit a higher risk of generating brain metastases. For these patients, available targeted therapies have demonstrated preventive benefits for brain metastases. However, these genomic alterations are present in a minority of patients affected by brain metastases.(B) Recent findings suggest that imaging data processed through the lens of radiomics and integration of the complex clinical history in addition to epigenomic marks could be used by artificial intelligence (AI) to define predictive biomarkers of brain metastasis. These preventive efforts would enable scoring of patients diagnosed with primary tumors with unknown risk of brain metastases. Some of these biomarkers have been validated in liquid blood biopsies. Subsequent development of these promising results might include preventive brain imaging to use effective local [i.e., stereotactic radiosurgery (SRS)] or systemic [i.e., immune checkpoint blockade (ICB)] therapies for asymptomatic brain metastases, which are easier to control. The term asymptomatic brain metastasis refers here to early metastatic seeding in the brain with absence of clinical symptoms, which may or may not be detected with magnetic resonance imaging (MRI), according to the size of the metastases. Thus, preventive therapies correspond to those intended to prevent metastatic seeding at all or to target early metastatic seeding before it develops into clinically symptomatic macrometastases. A pending aspect is the development of drugs with preventive purpose guided by the validation of biomarkers on liquid biopsy. Specific windows of opportunity should be used to effectively test compounds with a preventive purpose. For instance, relapse post-surgery could offer such a unique opportunity to generate clinical data on preventing brain metastasis relapse. Abbreviations: ALK, anaplastic lymphoma kinase; EGFR, epidermal growth factor receptor; HER2, human EGFR 2; SCLC, small-cell lung cancer; TNBC, triple-negative breast cancer.

The identification of a population of patients at high risk of developing CNS metastases – termed primary prevention – is essential to develop optimal, cost-effective, and targeted prevention strategies. A variety of predictive tools are being explored to support this goal, with rapid advances including machine learning and artificial intelligence that have helped to identify reliable biomarkers. Machine-learning approaches combining clinical data and radiomics appear particularly promising for both primary and secondary prevention; that is, strategies aimed at avoiding additional BrMs. In lung cancer patients, such tools have shown potential to select high-risk subgroups who may benefit from surveillance brain MRI [213,214]. In parallel, DNA methylation signatures derived from primary tumors were successfully used to build and validate models to predict CNS metastasis development using a risk probabilities nomogram, which integrates clinical data. Additionally, plasma methylomes were identified as accurate, noninvasive classifiers for early prediction and detection of BrMs [215,216] (Figure 4). While these findings are promising, the available predictive models still require independent validation in prospective, controlled clinical studies.

### Research efforts towards clinical prevention

Preclinical research provides a vast resource to functionally explore clinical correlations, validate biomarkers [101,217,218], and optimize therapeutic strategies for prevention [139]. However, a key challenge remains in translating these findings into clinically relevant scenarios. One promising approach is the use of window-of-opportunity trials, which allow therapeutic interventions to be tested in patients prior to standard treatments such as surgery. For example, patritumab deruxtecan, a potent ADC targeting HER3, is currently being investigated in a window-of-opportunity trial prior to craniotomy for BrM. The study aims to investigate the intra-and peritumoral microenvironment, assess safety, and explore potential biomarkers for intracranial response (NCT05620914)

To date, successful examples of primary or secondary CNS prevention have been limited to patients with very specific, well-defined truncal molecular alterations: shared between the primary tumor and BrMs, and for which a matched targeted therapy exists. In addition, a subpopulation of patients treated with ICB antibodies has shown reduced CNS progression (see earlier for more details). While these examples apply to relatively small patient populations, the results are nonetheless remarkable and demonstrate the feasibility of BrM prevention. For instance, in patients with stage III EGFR-mutant NSCLC, osimertinib significantly improved CNS PFS – 36% vs 9% with placebo – in those without progression during or following definitive platinum-based therapy [219]. Another example is the HER2CLIMB study, where the combination of tucatinib, trastuzumab, and capecitabine was able to delay the development of new BrMs by 45.1% compared with the placebo-combination group in patients with HER2^+^ metastatic breast cancer [220]. Tucatinib is now being evaluated for the secondary prevention of BrMs after local treatment of isolated intracranial progression on the trastuzumab-pertuzumab combination or TDM1 (NCT05323955).

Taken together, these advances highlight the potential of dedicated clinical studies, informed by data obtained in the preclinical setting and powered by innovative molecular and immunological strategies [221], to pave the way for new prevention approaches, Expanding efforts in both primary and secondary CNS prevention will be essential to shift the paradigm from reactive treatment to proactive, risk-adapted care.

## Concluding remarks

Together, the studies and strategies discussed in this review underscore a central theme: BrMs are not simply an extension of systemic disease, but a distinct biological and clinical entity. From the genomic and cellular features that govern brain tropism to the specialized microenvironmental and metabolic adaptations that enable survival and progression within the CNS, BrMs present unique vulnerabilities for therapeutic intervention. A more integrated approach – connecting high-resolution basic science with predictive tools, innovative models, therapeutic development, and patient-centered clinical design – is now within reach. As the field moves forward, continued investment in collaborative research frameworks, standardized clinical trial endpoints, and shared multimodal platforms will be key to translating these insights into clinical impact. Ultimately, by aligning mechanistic discovery with clinically actionable advances, we are poised to redefine how we detect, treat, and prevent BrMs (see Outstanding questions).

## Highlights

Since Volume I in 2018, the field has undergone transformative advances, reshaping both the scientific and the clinical landscape.

Volume II focuses on the pivotal discoveries that have propelled this progress, including omics strategies, the tumor microenvironment, metabolism, experimental models, technological advances, and therapeutic progress.

The past 7 years have seen a substantial increase in the number of clinical trials specifically including patients with brain metastases (BrMs), with emerging efforts in preventive strategies, antibody–drug conjugates, immunotherapy, and quality-of-life interventions.

Volume II captures the transition from foundational understanding to meaningful clinical applications. The advances we highlight here chart a path forward for innovative therapeutic strategies with the potential to significantly improve outcomes for patients with BrMs.

## Box 1. BrM consortia and epidemiology

Over the past decade, as the BrM field has grown and evolved, it has become apparent that discoveries and progress require a collective and multidisciplinary approach. Given the complexity of BrMs, collaborations among experts in basic and translational science, neurobiology, pharmacology, clinical trial design, and various medical specialties is essential to define the most pressing clinical challenges and to advance care to improve our patients’ outcomes.

In response to this need, several consortia have been established to drive preclinical discoveries, translate findings into clinical trials, and implement international studies for patients with BrMs. Notably, two major consortia founded in 2021 – RENACER, the Spanish National Network on Brain Metastases, and CIMARA, the Consortium of Intracranial Metastases of Academic Research – have been dedicated to improving patient outcomes through research and collaboration.

### RENACER: the Spanish National Network on Brain Metastases

RENACER is a collaborative network that includes multiple hospitals, a biobank, and research laboratories [222]. The network focuses on collecting fresh tumor samples from neurosurgeries, as well as liquid biopsies from patients with BrMs. These fresh samples are shipped, within 24 h in special media [222], to the Spanish National Cancer Research Centre (CNIO) Biobank. There they are processed into paraffin-embedded tissue, microtome tissue, DNA (including controls from peripheral blood), RNA, and organotypic cultures. These samples, along with clinical data, are available to both RENACER laboratories and the broader scientific community under the biobank framework.

Since its inception, RENACER has collected approximately 473 samples from 389 patients. Samples include 473 paraffin-embedded tissue blocks, 385 blood liquid biopsies, 29 cerebrospinal liquid biopsies, 86 organotypic cultures, 294 DNA samples, and 317 RNA samples that have been processed for genomic studies. Currently, sample availability for investigators external to the RENACER consortium does not include the whole-exome sequencing, transcriptomic data, or organotypic cultures, as these require an expanded ethical and legal framework for genomic data sharing, given strict patient privacy regulations. Thus, the current strategy under development is to create a Brain Metastasis Data Portal, which would fully comply with EU regulations to expand data-sharing capabilities [223]. Similar open-access approaches, such as the BrainTIME platform (Joyce laboratory, Switzerland), have been developed to share data from patient samples in a legally compliant and fully anonymized manner. While sharing organotypic cultures outside the RENACER network remains a challenge, as it will not be possible to track sample quality, this could be addressed through scientific collaborations with RENACER.

The impact of RENACER is already evident: in a short period of time, the network has contributed to five publications [47,73,138,139,222] and supported three clinical trials/studies [145] (NCT05635734, NCT05689619, Temporary ID: C/46/2023). RENACER has also incorporated MRI data, which are obtained during standard-of-care treatment. This enables noninvasive assessment of metastasis heterogeneity and differential response to treatment when multiple BrMs are present [224], and it is also the source of noninvasive biomarkers [225,226]. Additionally, the inclusion of neurocognitive assessments (for patients that sign the informed consent) addresses a critical gap in understanding the impact of BrM on cognition [227]. However, the absence of a standardized test, combined with limited resources for its implementation (i.e., access to clinical neuropsychologists) has prevented the establishment of a multi-institutional database. Therefore, RENACER is leveraging the ACS [74], an online resource that can be administered in nearly any hospital or even remotely at home, requiring only a computer, internet access, and an email account [228]. Together, these advances in neuroimaging and neurocognitive profiling will enable the establishment of a fully comprehensive database for BrM.

### CIMARA: a global effort to advance BrM research and treatment

CIMARA [229], also founded in 2021 by investigators at Duke University and MD Anderson Cancer Center in the USA, is an international consortium designed to unite investigators across disciplines and specialties to advance translational and clinical research in BrM. Its mission statement is as follows: ‘Clinical Investigators and Research Scientists who share the collective goal of improving outcomes for patients with central nervous system (CNS) metastases through awareness and coordination of multi-institutional clinical trials testing therapeutic agents for this unique patient population’. In addition to providing the international infrastructure to enhance discovery and development of BrM therapies, the CIMARA team is focused on harmonizing clinical trial endpoints regarding CNS metastases outcomes and providing access to common web-based forums for patients and healthcare providers to learn about clinical trial opportunities for patients with CNS metastases.

Currently, CIMARA has over 130 members, mostly across North America and Europe. Approximately 60% of its members are medical oncologists and neuro-oncologists, with the remaining 40% comprising basic science researchers, radiation oncologists, neurosurgeons, and patient advocates. Presently, the group meets virtually to address key questions in BrM research and design prospective and retrospective studies to tackle unmet needs in the field.

One of CIMARA’s early achievements was the publication of a collaborative statement defining ‘symptomatic’ melanoma BrM for use in clinical trials [230], with further work ongoing in this area. The consortium has prioritized the challenge of radiation therapy necrosis as the focus of its first large, multicenter, retrospective analysis: the BRACER study (‘Efficacy and safety of bevacizumab when combined with other systemic agents in the treatment of brain metastases radiation necrosis in the modern era’; Pro00114836). BRACER will evaluate the impact of the antiangiogenic therapy bevacizumab in managing radiation necrosis, while also examining clinical practice patterns and the natural history of this complex treatment-related toxicity. While some retrospective data exist [231,232], BRACER aims to provide modern clinical guidance on optimal radiation therapy management in the evolving era of novel therapeutics, including TKIs, ADCs, and advanced local therapy techniques such as laser interstitial thermal therapy (LITT) [233]. In parallel, CIMARA is partnering with the Breast International Group (BIG) on a similar retrospective analysis of radiation necrosis in breast cancer across Europe, exemplifying the consortium’s commitment to global, collaborative research efforts.

Future directions for CIMARA include investigating multimodality treatments, including the optimal timing (i.e., sequential or current treatment and/or order of therapies) of radiation and systemic therapies. Additional priorities include screening for breast cancer BrMs in high-risk patients, and defining optimal treatment paradigms for CNS metastases in the modern therapeutic landscape.

### The Vienna Brain Metastasis Registry: a large-scale retrospective effort

As the implementation of prospective clinical trials for patients with BrM has been historically challenging, we are also able to learn a tremendous amount of information from retrospective data registries focused on this patient population. The Vienna Brain Metastasis Registry [234] was developed as a single-institution database, with a high granularity of clinical data on the individual-patient level and collection of tumor tissue samples. Over more than three decades, this registry has collected data from over 6000 individual patients treated at the Medical University of Vienna.

While acknowledging the limitations of retrospective data analyses, the registry’s substantial sample size, the depth of clinical and biological data on the individual-patient level, and the long-term follow up offer valuable insights and research opportunities. Recent examples of analyses from the Vienna Brain Metastasis Registry highlight shifting trends in symptomatic presentation and treatment strategies [234,235], as well as the impact of targetable driver mutations and systemic inflammation on clinical presentation and prognosis [234,236–239]. Furthermore, the radiological database and biobank of the registry enables hypothesis-driven tissue-based research to identify prognostic and predictive biomarkers [185,240–243].

Beyond these observational data, retrospective analyses from the registry have influenced prospective clinical trial designs [244,245]. For example, early retrospective studies suggested that HER2-targeting ADCs could benefit patients with BrM [246]. This then led to the development of the prospective TUXEDO-1 trial, to incorporate T-DXd into international treatment recommendations [177,179,182]. Building on this approach, additional trials based on the same principle are ongoing (NCT05866432, NCT05865990). Overall, the example of the Vienna Brain Metastasis Registry shows that data generation from a retrospective, single center can be helpful to characterize clinical courses and their changes over time, identify prognostic and predictive biomarkers, and realize prospective clinical trial initiatives.

## Box 2. A future therapeutic pipeline to enhance patient quality of life?

Cognitive impairment is highly prevalent among patients with BrMs, resulting from both the tumor itself and various treatments for systemic and CNS disease. This impairment has a significant impact on the daily functioning of patients and their families [261]. In particular, neurocognitive dysfunction – encompassing domains such as memory, attention, executive function, and processing speed – has emerged as a key determinant of patient quality of life [66].

The therapeutic landscape for BrMs continues to expand, which includes options such as SRS, HA-WBRT, surgery, targeted therapy, immunotherapy, Optune, FLASH, LITT, and various combinations. These treatment modalities are layered onto the broad diversity of tumor types and patient needs, ranging from those with extensive treatment histories to those newly navigating a complex and evolving disease. Thus, it is clear that coordinating care and research is challenging.

Despite widespread recognition of the impact of cognitive impairment in patients with BrMs, cognitive outcomes remain underrepresented in clinical trials and routine care frameworks. Consequently, our understanding of the risk factors, trajectory, and presentation of cognitive decline in this population is limited. Not all patients experience treatment-related neurotoxicity to the same extent, and cognitive effects can vary significantly in both nature and timing. Why do some patients tolerate treatment better than others? Who is most at risk for cognitive impairment? Which factors offer protection against neurotoxicity? How can we better select patients for trials focused on cognitive protection?

A recent wave of research in cancer neuroscience has begun to elucidate how BrMs may impair neurocognition beyond mass effect or location-specific damage. Metastatic tumor cells can integrate into neural circuits, remodel synaptic connections, and disrupt neurotransmission, ultimately altering brain homeostasis and contributing to cognitive decline [68,262]. These mechanistic insights position neurocognition not only as a clinical outcome but also as a biological target for intervention.

Furthermore, machine-learning models can predict BrM subtypes, suggesting that each BrM model differentially affects brain microcircuits and their emerging oscillations [73]. This opens opportunities for future therapeutic development targeting the molecular mediators of cognitive impairment [73]. Drugs to revert or even prevent the impact of metastases on brain function will not necessarily target cancer cell proliferation or viability. Thus, scoring the efficacy of such therapies may require the implementation of additional strategies in the clinical management of patients, such as incorporating cognitive outcomes in research and clinical care moving forward.

Several options exist for cognitive assessment in oncology. A widely adopted approach is the brief core cognitive test battery proposed by the International Cognition and Cancer Task Force [263], which evaluates memory, executive function, and processing speed. These tests can be administered with minimal training and do not require on-site neuropsychological expertise. However, a potential barrier is the need for in-person assessment. Clinical trials often struggle with the completion of cognitive evaluations [264]. This may be due to non-compliance related to high symptom burden, but it also highlights the need for strong commitment of multidisciplinary clinical and research teams to prioritize and support cognitive assessments in research and clinical care.

One promising approach to improving cognitive assessment in clinical trials is the use of online cognitive assessments that allow patients to complete evaluations remotely and without supervision. An example of this strategy is the ACS [74]. The ACS is a fully online cognitive battery that has been extensively tested for its psychometric properties, including reliability and validity. It comprises computerized versions of seven classic neuropsychological tests assessing domains such as memory, attention, and executive function.

An added advantage of digital cognitive test batteries (at least those where researchers or clinicians have access to response-level data) is that high-resolution data allow deeper analysis of subtests, stimuli, and response patterns. This enables a more detailed understanding of specific cognitive processes beyond traditional face-to-face outcomes, thus improving the identification and characterization of impairment [265]. These insights enhance our understanding of how cancer and its treatments affect cognition and may help to define new targets for intervention [266].

In clinical care, routine assessment of self-reported health-related quality-of-life outcomes, such as fatigue and depression, is already standard practice in many hospitals. Integrating cognitive testing for selected patient groups into this existing infrastructure, alongside the routine inclusion of cognitive outcomes in BrM trials, would support the development of a neuro-focused precision-medicine approach. This would enable us to tailor treatments to individual patients and advance our ultimate goal: to prevent or control brain disease without causing toxicity and to extend survival while preserving cognitive function and quality of life.

## Box 3. Leptomeningeal metastasis (LM)

Cancer cells can spread to the cerebrospinal fluid (CSF)-filled areas surrounding the CNS, particularly the leptomeninges. When this occurs, it is termed LM. While various alternative terms have been used, including leptomeningeal carcinomatosis, carcinomatous meningitis, and meningeal carcinomatosis, LM is preferred. This terminology accurately reflects both the anatomical location (the leptomeninges, rather than other meningeal layers) and the broad range of malignancies that can infiltrate this space, beyond carcinomas alone.

The leptomeninges provide a distinct microenvironment, separate from the brain parenchyma. While LM shares some characteristics with parenchymal BrM, it is approximately tenfold less common, with an incidence ranging from 9% in breast cancer to 25% in SCLC [247–249]. LM differs mechanistically from parenchymal BrM in several key ways. Whereas the brain parenchyma is a densely cellular and electrically active environment composed of astrocytes, oligodendrocytes, neurons, and endothelial cells, the leptomeninges primarily consist of leptomeningeal fibroblasts [250] that comprise the pia and arachnoid layers, as well as the circulating CSF. The CSF is produced by the choroid plexuses – specialized secretory epithelial structures in the ventricular system. Although isotonic with brain interstitial fluid, the CSF is relatively hypoxic and deficient in glucose, proteins, lipids, amino acids, and micronutrients compared with serum [251].

Cancer cells may access the leptomeningeal space through multiple routes. They can enter via the arterial circulation through the choroid plexuses [252] or via the venous circulation through the venous plexuses surrounding the spinal cord [253]. Additionally, metastatic cancer residing in the bone marrow of the skull may reach the leptomeninges through bridging veins [254,255]. Another potential route involves cancer cells in the brain parenchyma invading perivascular spaces, which are in direct communication with the leptomeninges [39,256]. The chemotactic signals guiding these metastatic cells may be influenced by the neuroanatomical microenvironment and tumor-intrinsic factors, such breast cancer cells responding to and migrating towards sources of glial cell line-derived neurotrophic factor (GDNF) [254]. The factors determining which route a cancer cell takes remain unclear. However, cell-intrinsic properties dictated by the cell of origin likely play a significant role in this process.

Once within the leptomeningeal space, cancer cells can adopt two primary phenotypic states: adherent to the pial surface or floating in the CSF [257]. These two states exist in equilibrium, but the floating cells are particularly aggressive, representing the more lethal form of LM. Importantly, floating cells can be accessed through clinical CSF sampling, facilitating recent advances in single cell-based analyses that have provided insight into LM at the human scale [258,259].

In the absence of disease, the CSF is nearly acellular, containing only a small number of CD4^+^ T-cells. However, LM triggers a robust inflammatory response, characterized by an influx of cytokines and immune cells, predominantly macrophages/monocytes and T cells. This immune profile is quite different than that of the brain parenchyma. Notably, the antitumor response in the leptomeninges differs from that of the brain parenchyma: interferon gamma, produced by T cells, promotes dendritic cell maturation, sustains NK cell survival, and enhances tumor cell killing [260].

This inflammatory signaling also disrupts the blood–CSF barrier, enriching the CSF composition in ways that facilitate cancer cell survival and growth [252]. Moreover, inflammatory cytokines such as IL-8 and IL-6 induce gene expression changes in cancer cells, enabling them to outcompete other cells for scarce extracellular iron, further supporting their proliferation [259].

## Outstanding questions

### How can spatial and single-cell multiomic analyses be integrated to define BrM-tropic programs?

- What are the mechanisms linking cellular states with the TME?
- How can multimodal, single-cell atlases of matched primary tumors, BrMs, and longitudinal liquid biopsies be established?
- How can BrM datasets be expanded to include currently underrepresented tumor types?

### What are the bidirectional interactions between cancer cells and brain-resident cells?

- How do BrM–neuron interactions integrate into functional circuits, and how do they affect cognition and seizure susceptibility?
- How can neurocognitive assessments be systematically incorporated into clinical BrM studies?

### How can the immunosuppressive microenvironment be effectively targeted?

- What is the correlation between spatial organization, TME diversity, and therapeutic response?
- How can translational studies targeting immunosuppressive mechanisms be accelerated and expanded?
- How can spatial profiling and multiplexed immune phenotyping be incorporated into clinical trials?
- Which novel strategies (e.g., brainpenetrant T cell engagers, localized cytokine delivery, intrathecal immunotherapy) should be prioritized?

### How can predictive and preventive tools be built into standard care?

- How can efforts to predict CNS relapse be prospectively validated in large, multi-institutional studies?
- How should predictive tools be incorporated into adjuvant trials for high-risk cancer patients?
- What is the feasibility, cost-effectiveness, and patient impact of risk-adapted MRI surveillance and CNS-directed prophylaxis in molecularly stratified populations?

### How can clinically relevant prevention paradigms be established and refined?

- How can prevention strategies be expanded beyond HER2^+^ or EGFR-mutant tumors?
- What actionable brain-tropic phenotypes exist in the absence of truncal mutations?
- How can window-of-opportunity trials in high-risk patients enable early CNS biomarker discovery?
- Should preventive strategies include immunomodulatory or microenvironment-targeting agents to alter metastatic seeding?

### How can BrM be modeled in biologically and clinically accurate contexts?

- How can GEMMs and syngeneic models be developed to reflect the full metastatic cascade?
- How can longitudinal sampling and standard-of-care modalities (e.g., SRS, surgery) be incorporated?
- How can human-based models be integrated to dissect cell–cell interactions and explore therapies?
- How can dynamic readouts be leveraged to provide a functional understanding of tumor–host interactions?

### How can patient-centered endpoints and quality of life be prioritized?

- How can cognitive preservation and quality of life become central endpoints in trial design?
- How can imaging, neurocognitive scores, and omics data be combined in prognostic and response analyses?
- Can CNS-directed systemic agents with parallel cognitive monitoring in patients with CNS-only progression demonstrate that disease control translates into functional benefit?
